# Fabrication and Characteristics of Porous Hydroxyapatite-CaO Composite Nanofibers for Biomedical Applications

**DOI:** 10.3390/nano8080570

**Published:** 2018-07-26

**Authors:** Shiao-Wen Tsai, Sheng-Siang Huang, Wen-Xin Yu, Yu-Wei Hsu, Fu-Yin Hsu

**Affiliations:** 1 Graduate Institute of Biomedical Engineering, Chang Gung University, Taoyuan City 33302, Taiwan; swtsai@mail.cgu.edu.tw; 2 Department of Orthopedic Surgery, Chang Gung Memorial Hospital, Linkou 33305, Taiwan; 3 Department of Periodontics, Chang Gung Memorial Hospital, Taipei 10507, Taiwan; 4 Department of Bioscience and Biotechnology, National Taiwan Ocean University, Keelung City 20224, Taiwan; mm0070360@gmail.com (S.-S.H.); andy54861@yahoo.com.tw (W.-X.Y.); qazwest74@gmail.com (Y.W.H.)

**Keywords:** sol-gel, electrospinning, hydroxyapatite, nanofiber, antibacterial

## Abstract

Hydroxyapatite (HAp), a major inorganic and essential component of normal bone and teeth, is a promising biomaterial due to its excellent biocompatibility, bioactivity, and osteoconductivity. Therefore, synthetic HAp has been widely used as a bone substitute, cell carrier, and delivery carrier of therapeutic genes or drugs. Mesoporous materials have attracted considerable attention due to their relatively high surface area, large pore volume, high porosity, and tunable pore size. Recently, mesoporous HAp has also been successfully synthesized by the traditional template-based process and has been demonstrated to possess better drug-loading and release efficiencies than traditional HAp. It is widely accepted that cell adhesion and most cellular activities, including spreading, migration, proliferation, gene expression, surface antigen display, and cytoskeletal functioning, are sensitive to the topography and molecular composition of the matrix. The native extracellular matrix is a porous, nanofibrous structure. The major focus of this study is the fabrication of porous hydroxyapatite-CaO composite nanofibers (p-HApFs) and the investigation of its drug-release property. In this study, nanofibers were prepared by the sol-gel route and an electrospinning technique to mimic the three-dimensional structure of the natural extracellular matrix. We analyzed the components of fibers using X-ray diffraction and determined the morphology of fibers using scanning and transmission electron microscopy. The average diameter of the nanofibers was approximately 461 ± 186 nm. The N_2_ adsorption–desorption isotherms were type IV isotherms. Moreover, p-HApFs had better drug-loading efficiency and could retard the burst release of tetracycline and maintain antibacterial activity for a period of 7 days. Hence, p-HApFs have the potential to become a new bone graft material.

## 1. Introduction

Bone is a natural inorganic–organic composite consisting of collagen fibrils containing well-arrayed apatite nanocrystals. Hydroxyapatite [HAp, Ca_10_(PO_4_)_6_(OH)_2_] is chemically similar to the inorganic component of the bone matrix, which has led to extensive research efforts to use this material as a bone substitute in both orthopedic and dental fields. Recently, HAp has been used in range of biomedical applications, such as matrices for drug release and bone tissue engineering scaffold materials. 

HAp can be synthesized through a variety of well-developed techniques, such as the sol-gel process [1], the wet-chemical reaction [2], the solid-state reaction [3] and the chemical vapor deposition [4]. The synthesis of HAp using the sol-gel method consists of the molecular-level mixing of calcium and phosphorus precursors under significantly milder conditions. Hence, a sol-gel synthesis of HAp has recently attracted considerable attention. 

The sol-gel synthesis technique is also used in conjunction with various spinning techniques to fabricate HAp fibers [5]. Electrospinning is an easy and simple method that utilizes electrical fields to fabricate nano- to microfibers from a polymer solution. The topological structure of the electrospun matrix closely mimics the dimensions of the natural extracellular matrix (ECM). The ECM plays a key role in triggering intracellular signaling cascades for tissue regeneration. Thus, the development of new ECM substitutes has attracted a wide attention as a scaffold for tissue engineering. Franco et al. successfully fabricated HAp nanofibers by combining electrospinning and a non-alkoxide sol-gel system [6]. Pasuri noted that electrospun HAp nanofibers do not activate macrophages in vitro and can be resorbed by human osteoclasts [7].

Recently, much attention has been attracted by HAp as a drug delivery carrier for the loading and delivery of therapeutic agents, such as proteins, growth factors, genes, and drugs [8,9,10]. Mesoporous HA nanoparticles possess a higher drug-loading capacity and drug-release efficiency than HAp nanoparticles as a result of their large surface areas and high pore volumes [11]. The synthesis of mesoporous HAp nanoparticles using template reagents, such as Pluronic P123 [12], cetyltrimethylammonium bromide (CTAB) [13], and Pluronic F127 [14], is the most well-known method. In the synthesis of mesoporous HAp, the self-assembly of inorganic HAp phases and template reagents followed by template removal can produce the mesoporous structure.

Münchow [15] incorporated CaO nanoparticles into electrospun matrices and demonstrated improved cell viability and osteogenic differentiation. Hence, the aims of this study were to fabricate and characterize a nanofibrous structure of porous hydroxyapatite-CaO (p-HApFs) by utilizing an electrospinning process based on a sol-gel precursor and to evaluate the release profiles and antibacterial activity of tetracycline from p-HApFs.

## 2. Materials and Methods 

### 2.1. Synthesis and Characterization of Porous Hydroxyapatite Nanofibers

Mesoporous hydroxyapatite nanofibers were synthesized using poly(ethylene glycol)-block-poly(propylene glycol)-block-poly(ethylene glycol) (P123, Sigma-Aldrich, St. Louis, MO, USA) as the structure-directing agent. Briefly, 1.0 g of P123 and 6.172 mL of triethyl phosphite (TEP, Merck, Germany) were mixed in a 10 mL ethanol aqueous solution (50% *v*/*v*), which was then continuously stirred to form a clear solution. Ca(NO_3_)_2_ (14 g, Sigma-Aldrich, St. Louis, MO, USA) was dissolved in 10 mL of absolute ethanol under magnetic stirring at room temperature. The calcium nitrate solution was slowly added under stirring to the above P123/TEP solution to form a precursor solution. The precursor solution was tightly capped and placed in an oven at 60 °C for 12 h. Then, 1.5 g poly(vinyl pyrrolidone) (PVP, Sigma-Aldrich, St. Louis, MO, USA), 0.45 g P123 and 7 mL absolute ethanol were prepared and incorporated into the 3 mL precursor solution to obtain a transparent mixture solution. The mixture solution was placed inside a plastic syringe fitted with a stainless needle (18 G, inner diameter = 0.838 mm) and then inserted into a syringe pump to supply a steady flow rate (1.27 mL/h). An electrical field (1.3 kV/cm) was applied between the needle and an aluminum substrate (grounded collector) using a high-voltage power supply (SL 60, Spellman, New York, NY, USA). The polymer solution, which formed a Taylor cone upon exit, was collected on an aluminum substrate in the form of nonwoven nanofiber structures and was then calcined at different temperatures (600 °C, 800 °C and 1000 °C) and environments (air and nitrogen) to obtain the calcined p-HApFs nonwoven structures.

### 2.2. Characterization of the p-HApF 

The p-HApFs morphology was observed using scanning electron microscopy (SEM, Hitachi S-4800, Tokyo, Japan), which operated at an accelerating voltage of 15 kV The average fiber diameter of the p-HApFs was analyzed by image analysis software (Image-Pro Express Version 6.0, Media Cybernetics, Rockville, MD, USA) based on the SEM images. Moreover, the p-HApFs pore structure was observed using transmission electron microscopy (TEM, JEOL JEM-2100, Tokyo, Japan) at an accelerating voltage of 100 kV. Nitrogen adsorption–desorption measurements were used to obtain the Brunauer-Emmett-Teller (BET) specific surface area, and the pore size was also analyzed (Micromeritic ASAP 2020 instrument, Norcross, GA, USA). The phase of p-HApFs was characterized by X-ray diffraction (XRD, Bruker D2-Phaser, Madison, WI, USA) with a copper target. Powder diffraction patterns were acquired over 2-theta, ranging from 20 to 60° with a step size of 0.04°. The functional groups of p-HApFs were analysed with Fourier transitioned infrared spectroscopy (FTIR, Bruker tensor II, Madison, WI, USA). The spectra were recorded from 4000 to 400 cm^−1^ wave number with a resolution of 2 cm^−1^.

### 2.3. In Vitro Study of Drug Loading and Release

Tetracycline (TC) was selected as the model drug for the evaluation of drug loading and release. p-HApFs (10 mg) was added to a TC aqueous solution (5 mg/mL, 10 mL) and stirred for 24 h. After the loading procedure, the amount of TC adsorbed onto p-HApFs, which calcined at N_2_ atmosphere 800 °C, was calculated by determining the difference in the TC concentration before and after loading. The TC concentration was analyzed at a wavelength of 360 nm using a UV-Vis spectrophotometer (Ultrospec 1100 Pro, Amersham Biosciences, Piscataway, NJ, USA). 

The drug contents and loading efficiency were calculated according to the following formulas:

Drug content (*w*/*w*) = weight of the TC in the p-HApFs/weight of the p-HApFs

Loading efficiency (%) = (weight of the TC in the p-HApFs/initial weight of the p-HApFs) × 100%

After loading, the p-HApFs were dried using a lyophilization process. TC-loaded p-HApFs (20 mg) were added to a phosphate-buffered saline (PBS) solution (2 mL) and agitated in a horizontally shaking bath at 37 °C. The release medium was withdrawn and replaced with fresh PBS at each measurement. The mechanism of drug release was analyzed by fitting the experimental data to equations describing different kinetic orders. Linear regression analyses were performed for zero-order [M_t_/M_0_ = K_0_·t] and first-order [ln(M_0_ − M_t_) = K_1_·t] kinetics, as well as the Higuchi [M_t_/M_0_ = K_H_·t^1/2^] model, where K is the kinetic constant and M_t_/M_0_ is the fraction of TC released at time t. The best-fitted model was assessed on the basis of the correlation coefficient (r^2^). The drug release data were further analyzed with the Ritger-Peppas equation [M_t_/M_∞_ = k_r_·t^n^], where Mt is the amount of drug released at time t, M_∞_ is the amount of drug released at time ∞, and k_r_ and n represent the release rate constant and release exponent, respectively. 

### 2.4. Antibiotic Activity against Staphylococcus aureus and Pseudomonas aeruginosa

The antibiotic activity of TC in the in vitro release study was assessed against a Gram-positive bacterial strain, *Staphylococcus aureus*, and a Gram-negative bacterial strain, *Pseudomonas aeruginosa*, using the antibiotic elution samples from a turbidity assay. TC (500 μL) eluated during the in vitro release study or from PBS (as the control group) was added to the inoculum containing 500 μL of the 2× nutrient broth solution and 100 μL of bacteria in 5-mL test tubes. After 24 h of incubation, the optical density (OD) of the culture medium was measured using a spectrophotometer at a wavelength of 600 nm. The relative antibacterial activity (R%) was calculated as follows:

R% = [(OD_growth medium_ − OD_released tetracycline_)/OD_growth medium_] × 100%

## 3. Results and Discussions

**Figure 1:**
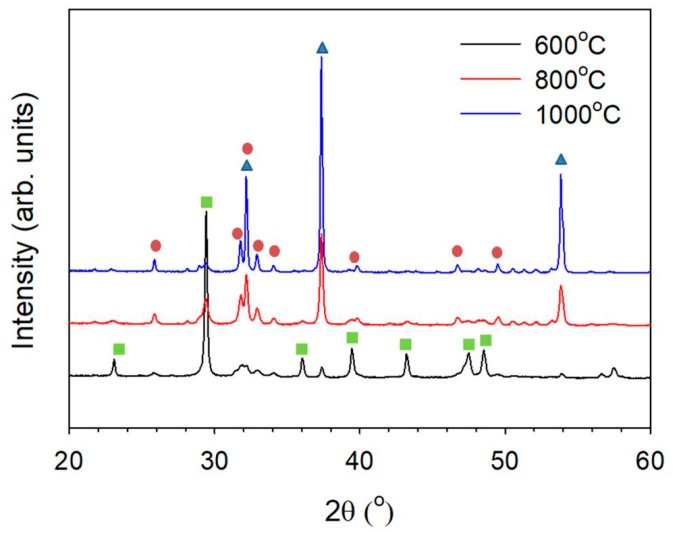
XRD pattern of the fibers calcined at different temperatures under an air atmosphere. (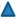: CaO, PDF 70-4068; 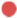: hydroxyapatite, PDF 84-1998; 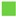: CaCO_3_, PDF 85-1108).

All of the electrospun nanofibers were calcined at three different temperatures, namely, 600, 800 and 1000 °C, to study the crystal phase structure. Figure 1 shows the XRD pattern for the nanofibers calcined at different temperatures. The electrospun fibers were mostly crystalline calcium carbonate at 600 °C. The amount of CaO increased when the calcination temperature increased, which was due to the breakdown of calcium carbonate that formed calcium oxide, accompanied by the evolution of CO_2_. The HAp phase formed at 800 °C.

**Figure 2:**
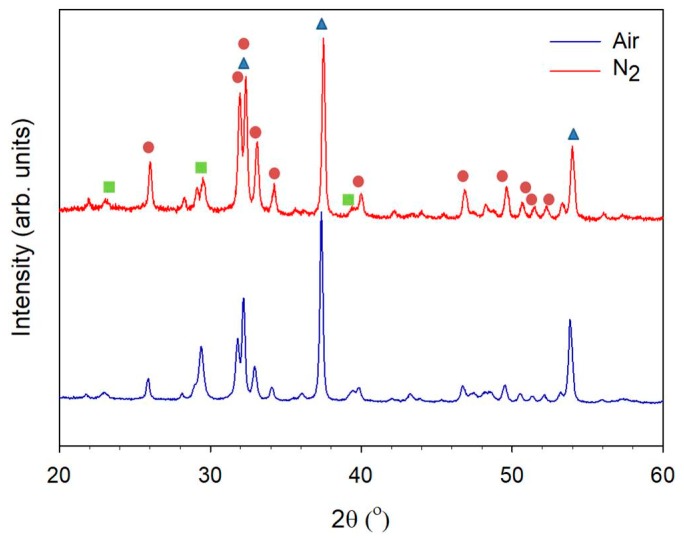
XRD pattern of the fibers calcined at 800 °C under various calcination atmospheres. (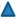: CaO, PDF 70-4068; 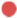: hydroxyapatite, PDF 84-1998; 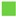: CaCO_3_, PDF 85-1108).

Figure 2 shows the XRD pattern for the nanofibers calcined under air and nitrogen atmosphere at 800 °C. The main crystal phase comprised HAp and CaO. However, the amount of HAp was higher under the nitrogen than under the air atmosphere. The amount of dissolved ambient CO_2_ can be limited by performing the reaction under a nitrogen atmosphere [16]. Hatzistavrou [17] fabricated hydroxyapatite-CaO composites using the sol-gel method and found that the presence of CaO accelerated the formation of carbonate hydroxyapatite in simulated body fluid. This was due to the dissolution of the CaO phase that rapidly formed carbonate hydroxyapatite. Hence, HAp/CaO composites have been used as scaffolds with tuneable properties by varying the composition. 

**Figure 3:**
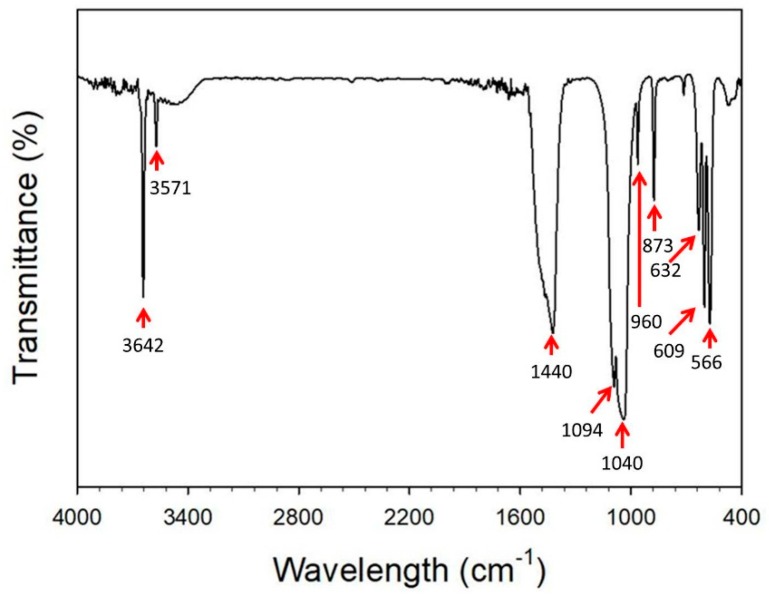
FTIR spectra of the fibers calcined at 800 °C under an N_2_ atmosphere.

Figure 3 shows a Fourier transform infrared (FTIR) characteristic spectrum for the nanofibers that were calcined at 800 °C under a nitrogen atmosphere. Peaks at approximately 566 and 609 cm^−1^ are due to the bending vibration of the P-O bond in PO_4_^3−^ [18]. The bands at approximately 960 and 1000~1100 cm^−1^ are associated with the stretching modes of the PO_4_^3−^ bonds in Hap [19]. The characteristic bands at approximately 873 and 1440~1470 cm^−1^ are attributable to the CO_3_^−2^ group. Taherian et al. [20] pointed out CO_3_^2−^ ions may form due to the incomplete pyrolysis of organic compounds, or the absorption of CO_2_ from the atmosphere. Bilton et al. [21] pointed out that the evaporation of unreacted triethyl phosphite from the sol or gel could constitute to the presence of CaCO_3_. CaCO_3_ decomposes to CaO after calcination at 800 °C

The dissolution of CO_2_ from the atmosphere occurs by the following reaction:

CO_2_ (g) + 2 OH^−^ (aq) → CO_3_^2−^ (aq) + H_2_O (l)

The characteristic peaks observed at 632 and 3571 cm^−1^ are attributable to the respective hydroxyl functional group (-OH) deformation vibration and stretching vibrations of HAp [22]. Additionally, a sharp peak was observed at 3642 cm^−1^, which confirms the formation of the CaO phase [23]. 

**Figure 4:**
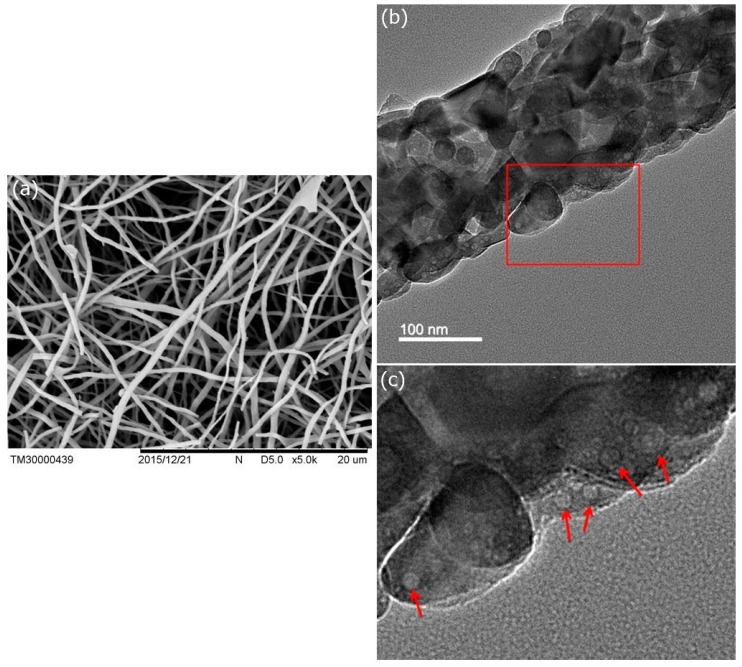
(**a**) SEM and (**b**) TEM micrographs of the nanofibers after heat treatment at 800 °C under an N_2_ atmosphere. (**c**) an enlarged graph in red square of (**b**). The red arrows in (**c**) indicates mesopores within the nanocrystals.

The morphology and structure of the p-HApFs were observed under SEM and TEM. Figure 4a shows the SEM image of the p-HApFs. The average diameter of the p-HApFs was 461 ± 186 nm. Figure 4b shows the TEM image of the p-HApFs. The TEM image showed that the nanofiber was composed of a number of nanocrystals and clearly revealed the existence of mesopores within the nanocrystals. The p-HApFs did not exhibit an ordered orientation of the mesopores, which generally had a random arrangement. 

**Figure 5:**
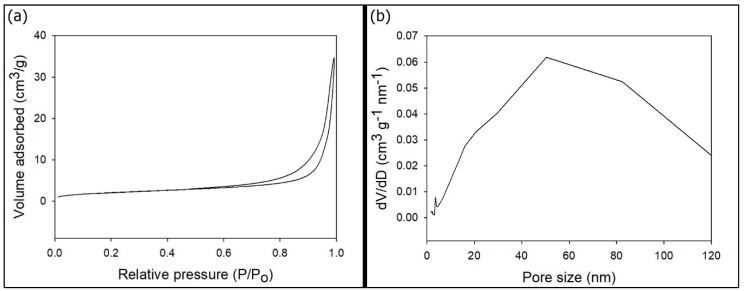
(**a**) N_2_ adsorption–desorption isotherm and (**b**) pore size distribution curve of p-HApFs.

The nitrogen adsorption–desorption isotherms of the p-HApFs were type IV hysteresis loops, which are typical for mesoporous materials (shown in Figure 5a). The specific surface areas of the p-HApFs calculated from the BET equation were 7.2 m^2^/g. The pore size distribution was plotted according to the BJH nitrogen desorption model as shown in Figure 5b. The average pore size of the p-HApFs calculated from the BJH equation was approximately 28 nm. Figure 5b shows a broad peak ranging from 10 to 130 nm, centered at approximately 50 nm, which suggests that most pores had a size of approximately 50 nm; however, the pore sizes were not uniform. 

**Figure 6:**
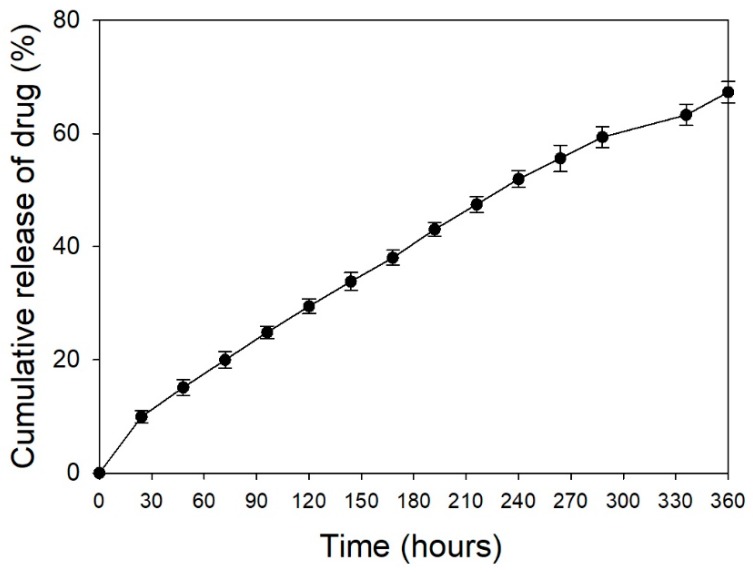
In vitro cumulative tetracycline (TC) release from TC-loaded p-HApFs.

The amount of TC loaded within the p-HApFs was 8.83 ± 0.09 mg/10 mg (TC/p-HApFs). The loading efficiency of TC was 88.34 ± 0.89% (*w*/*w*). The cumulative drug-release curve as a function of time for TC release from the TC-loaded p-HApFs was analyzed in triplicate (shown in Figure 6). The drug-release data indicated that a no-burst release phenomenon occurred at the initial step. The TC release from the TC-loaded p-HApFs was steady and slow over a period of 14 days. The drug-release data were analyzed using different kinetic models: zero-order, first-order, Higuchi, and Korsmeyer-Peppas. The best fit with the highest correlation coefficient (*r*^2^) was obtained using the first-order equation (*r*^2^ = 0.996), followed by the Higuchi model (*r*^2^ = 0.941), and zero-order equation (*r*^2^ = 0.983). The diffusion exponent *n*-value of the Korsmeyer–Peppas model was 0.75, which indicates anomalous diffusion or both diffusion- and erosion-controlled drug release [24].

**Figure 7:**
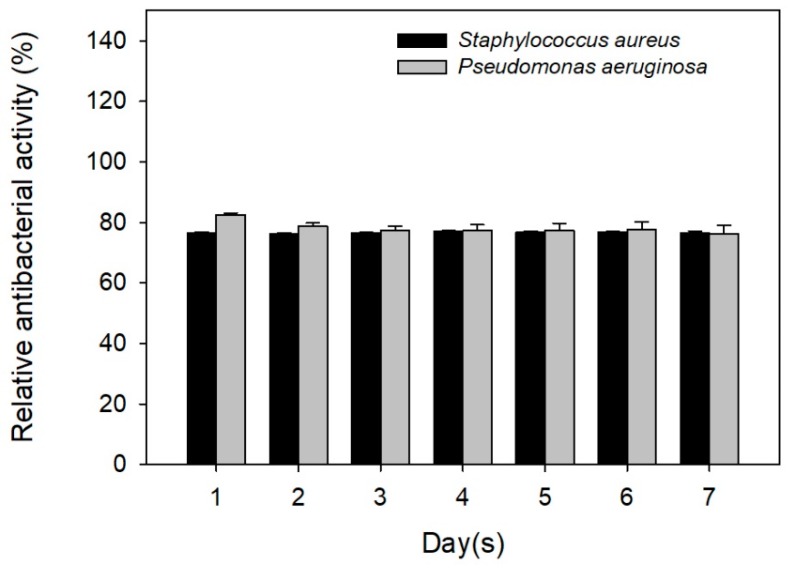
Susceptibility trend of the released tetracycline against *Staphylococcus aureus* and *Pseudomonas aeruginosa*.

To clarify the antibacterial activity of TC released from the TC-loaded p-HApFs, the release solution was used to cultivate bacterial strains. As shown in Figure 7, the release solution had a strong ability to inhibit bacterial growth, even on day 7.

## 4. Conclusions

A mesoporous hydroxyapatite nanofibrous matrix to mimic the three-dimensional structure of the natural extracellular matrix was successfully fabricated by an electrospinning process based on a sol-gel precursor. p-HApFs exhibited a high drug-loading efficiency and could retard the burst release of TC, as well as maintain antibacterial activity over a period of 7 days. Hence, it is anticipated that p-HApFs have the potential to be used as drug delivery carriers and as bone graft substitutes.
